# Primary Extranodal Marginal Zone B Lymphoma of the Uterus: A Case Report and a Review of the Literature

**DOI:** 10.1002/cnr2.70743

**Published:** 2026-11-14

**Authors:** Valeria Matera, Laura Bandiera, Manuela Bramerio, Maria Deodato, Carmela Quatrale, Liliana Marchetta, Mariachiara Palucci, Tommaso Bignardi, Maria Lieta Interdonato, Giuseppe Franco Cicala, Federico Spelzini, Lorenzo Ceppi

**Affiliations:** ^1^ Department of Medicine and Surgery, Obstetrics and Gynaecology Milano‐Bicocca University Monza Italy; ^2^ Department of Obstetrics and Gynaecology, ASST Grande Ospedale Metropolitano Niguarda Milano Italy; ^3^ Pathology Unit, ASST Grande Ospedale Metropolitano Niguarda Milan Italy; ^4^ Division of Haematology, ASST Grande Ospedale Metropolitano Niguarda Milan Italy; ^5^ Department of Obstetrics and Gynaecology, Humanitas San Pio X Milan Italy; ^6^ Università Vita‐Salute San Raffaele, Milan, Italy; Department of Experimental Radiology, IRCCS Ospedale San Raffaele Milan Italy; ^7^ Gynecologic Oncology Unit Fondazione IRCCS Istituto Nazionale Dei Tumori Di Milano Milan Italy

**Keywords:** Ann Arbor staging system, extranodal non‐Hodgkin lymphoma, female genital tract, immunotherapy, marginal zone lymphoma, uterus

## Abstract

**Background:**

Non‐Hodgkin's Lymphomas (NHL) of the female genital tract are extremely rare. Due to their low incidence, diagnosis can be challenging, particularly in advanced stages where establishing the primary nature of the disease is difficult in the absence of specific guidelines.

**Case:**

We report the case of a 65‐year‐old woman who presented with dyspnea and massive lower limb oedema. Imaging revealed a pelvic mass suspected to originate from the uterus and adnexa, extending into the cervical canal and vaginal walls, which appeared irregularly thickened and heterogeneous. Biopsies of the vaginal and cervical tissues revealed fragments of mature B‐cell lymphoma, consistent with a marginal zone histotype. The patient had a diagnosis of Stage IV extranodal marginal zone lymphoma (EMZL) of mucosa‐associated lymphoid tissue (MALT). She was enrolled in an experimental trial and demonstrated a partial response to the treatment.

**Conclusions:**

We reviewed previously reported cases in the literature, focusing on the clinical presentation and characteristics of this rare disease. Due to its non‐specific symptoms, a multidisciplinary approach is essential for timely diagnosis and appropriate treatment. Enrolment in an experimental study should be considered to advance knowledge and improve management.

## 1 Introduction

Lymphoma is a well‐known hematological malignancy, classified into Hodgkin lymphomas and the more common non‐Hodgkin lymphomas (NHL) [1]. When lymphoma arises from an organ or tissue outside the lymph nodes or spleen, it is classified as primary extranodal lymphoma (PEL), which accounts for 25%–40% of all primary lymphoma cases [2].

The involvement of the female genital tract in PEL is exceedingly rare, comprising approximately 1% of all PEL, with 300 reported cases of primary cervical lymphoma (PCL) [3], that represents 0.008% of all cervical cancers [4].

The definition of primary lymphomas of the female genital tract (gynPELs) has been controversial for years. Many relevant reports lack clear references to accepted classifications, specific markers, or molecular techniques necessary for reliable diagnosis. The WHO's 2016 revision of the classification of lymphoid neoplasms does not directly define uterine PELs [5]. In a study by Kosari et al. [6] 186 patients were analyzed using the 2003 WHO classification criteria to distinguish between primary and secondary female genital lymphomas, with definition of primary if the following criteria were met: 1. disease confined to the female genital tract at diagnosis; 2. no distant disease was present; 3. peripheral blood, bone marrow and remote organs were not involved within a six‐month period. Malignant lymphomas were considered to involve the genital tract secondarily if the disease, as demonstrated by biopsy, preceded the genital tract tumor or if the tumor was simultaneously present in lymph nodes and/or extragenital organs [6].

Krol et al. [7] proposed a broader definition of PEL, including all patients presenting with lymphoma that apparently originated at an extranodal site, also in the presence of diffuse disease, as long as the extranodal component was clinically more relevant. Their analysis illustrates the selection bias introduced when a strict definition of primary extranodal NHL excludes cases with disseminated disease from further analysis.

The treatment of extranodal lymphoma depends on various factors, including the subtype, stage of the disease, and the patient's overall health. Conventional treatments include chemotherapy, radiation therapy, targeted therapy, and immunotherapy. Currently, there are no randomized clinical trials or specific guidelines for the treatment of gynPELs. However, due to the very low incidence and the challenges in establishing a definitive diagnosis through imaging techniques, most of these tumors, particularly those located in the uterus or ovaries, are initially treated with radical surgery. Following the diagnosis of gynPELs, a standard chemotherapy regimen, such as R‐CHOP, typically used for aggressive or advanced NHL, is usually administered [8].

To illustrate the characteristics and challenges of gynPELs, we present the case of a woman with disseminated uterine lymphoma, detailing the associated diagnostic and therapeutic strategies.

## 2 Case Report

We report the case of a 65‐year‐old woman who presented to the Emergency Department of Niguarda Hospital in Milan in January 2023 with acute dyspnoea and significant lower extremity oedema. Her family history was notable for breast cancer in her mother, while her personal medical history included autoimmune hypothyroidism and two caesarean deliveries. Ultrasound assessment excluded deep venous thrombosis and revealed a pelvic‐abdominal hypoechoic solid mass with irregular margins, measuring approximately 160 × 110 × 130 mm, exhibiting rich vascularization on color Doppler imaging. The mass extended into the pelvis, causing bladder compression. Additionally, ascites and enlarged abdominal lymph nodes were observed. These findings raised suspicion of a malignant ovarian tumor, prompting further investigation with a chest‐abdomen CT scan for staging (Figure 1). The CT scan report identified thoracic venous thrombosis involving the right external jugular vein, subclavian vein, and the confluence into the superior vena cava. A massive right pleural effusion was also noted, with subtotal right lower lobe lung atelectasis. Abdominal study identified a vascularized pathological pelvic mass measuring 190 × 160 × 140 mm, involving the uterus and adnexa, with compression of the sigmoid colon and rectum, with no signs of bowel obstruction. Additionally, a third‐degree right hydroureteronephrosis was observed, with multiple confluent nodal enlargements located bilaterally in the para‐aortic and iliac‐obturator regions, as well as in the inguinal areas. At the gynecological examination, a solid mass was identified, occupying the entire pelvic cavity and extending up to the transverse umbilical line. The external genitalia appeared normal, while the vaginal walls and cervix were markedly oedematous and uniformly thickened. The rectovaginal space was filled with solid tissue. The patient was admitted to the gynecology department for further diagnostic evaluation. Tumor markers were assessed, with all results negative except for CA‐125, which was elevated at 600 U/mL (Table 1). A thoracentesis was performed, yielding 2000 cc of clear pleural fluid. Subsequent culture analyzes and cytological examination for malignant cells were negative.

**FIGURE 1:**
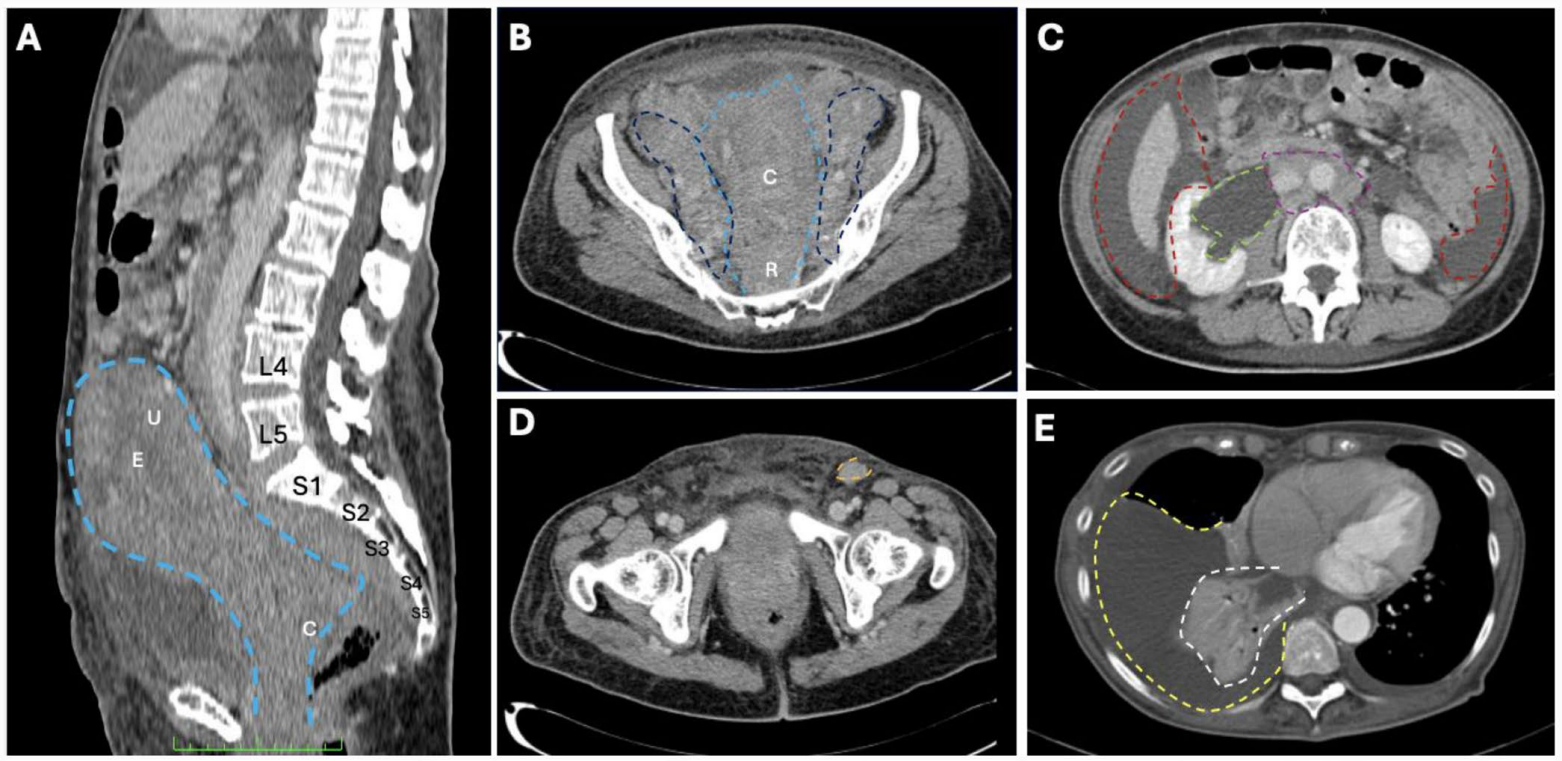
Chest‐abdomen CT scan at the emergency department (ED). (A) Shows a massive enlargement of the uterus (U) and cervix (C), delineated by light blue dashed lines, with a dilated endometrial cavity (E). Notably, the upper border of the uterine fundus nearly reaches the superior endplate of the L4 vertebral body. (B) Demonstrates a pathological pelvic mass involving the uterus and cervix (C), which encases the rectum (pelvic mass is outlined by light blue dashed lines) without evidence of mechanical bowel obstruction. Multiple confluent lymphadenopathies are observed bilaterally in the iliac‐obturator regions (dark blue dashed lines). (C) Highlights enlarged lymph nodes in the para‐aortic region (purple dashed lines), right‐sided grade III hydroureteronephrosis (green dashed lines), likely due to ureteral compression, and the presence of ascitic fluid (red dashed lines). (D) Shows lymphadenopathies in the inguinal regions, with one clearly marked on the left side (orange dashed lines). (E) Reveals a massive right pleural effusion (yellow dashed lines) associated with subtotal atelectasis of the right lower lobe (white dashed lines).

**TABLE 1:** Tumor markers.

	01/2023	03/2023	06/2023
CA‐ 125 (0–35 U/mL)	600	432	150
CEA (0–5 ng/mL)	0,6	/	/
CA 19.9 (0–37 U/mL)	4,6	/	/
AFP (0–7 ng/mL)	1,1	/	/
LDH (135–225 U/L)	180	232	312

*Note:* Tumor markers at the time of diagnosis (January 2023) and at one and 4 months following the initiation of treatment, respectively.

T2‐weighted MRI (Figure 2) revealed an enlarged uterus with a distended endometrial cavity, indistinguishable from the neoplastic tissue that filled the pelvis (22 × 8 cm). The neoplastic findings appeared to originate from the uterus, suggestive of a malignant mesenchymal origin. Anteriorly, the uterus was inseparable from the bladder, which exhibited thickened walls. Posteriorly, similar pathological tissue was observed, involving the rectum and sigmoid colon without evidence of infiltration. The right ovary, of normal size, was displaced to the iliac fossa, while the left ovary was unrecognizable. Several confluent lymph nodes with pathological characteristics were noted around the abdominal aorta, inferior vena cava, and iliac vessels.

**FIGURE 2:**
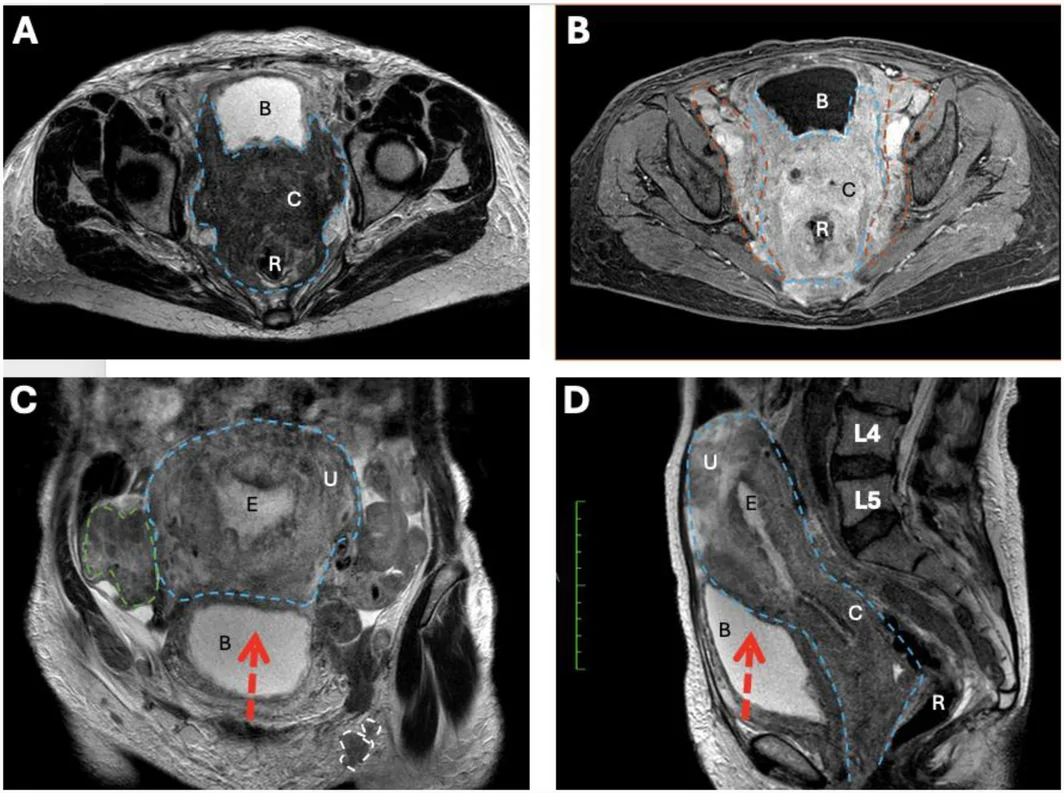
MRI scan of pelvis. (A, C, and D) are multiplanar T2‐weighted sequences, while Figure B is a T1‐weighted fat‐saturated sequence acquired after the intravenous injection of contrast medium. (A) Shows a hypointense neoplastic mass (light blue dashed lines) that appears to originate from the uterus and involves the cervix (C), occupying the entire pelvic space and extending anteriorly toward the bladder (B) and posteriorly toward the rectum (R). (B) Acquired on a similar plane to A, demonstrates avid contrast enhancement of the pathological mass (light blue dashed lines) following gadolinium administration. The lesion remains inseparable from both the bladder (B) and the rectum (R) and is associated with multiple confluent and enlarged lymph nodes bilaterally in the iliac and obturator chains (orange dashed lines). (C, D) Show a markedly enlarged uterus (U), outlined by light blue dashed lines, with a distended endometrial cavity (E) and anterior adhesion of the uterus to the posterior wall of the bladder (red dashed arrows). C also depicts the right ovary displaced superiorly into the right iliac fossa (green dashed lines), likely due to mass effect, as well as left inguinal lymphadenopathies (white dashed lines). (D) The uterine enlargement is clearly visible, extending up to the superior border of the L4 vertebral body. The mass appears inseparable from the walls of both the bladder (B) and the rectum (R).

This pathological tissue also compressed the lumbar segment of the right ureter, resulting in ipsilateral hydronephrosis. In the suspect of a metastatic disease with gynecologic origin, the multidisciplinary board proposed to meet the case, reviewed in a multidisciplinary meeting, where it was decided to proceed with a biopsy to determine the nature of the neoplasm. The vaginal wall and cervix were biopsied as the most accessible sites for tumor tissue representation. Histological examination revealed fragments of mature B‐cell lymphoma, specifically compatible with a marginal zone lymphoma histotype (WHO 2022 classification). The lymphomatous population was characterized by a diffuse infiltrate of small‐sized cells with irregular nuclei and clear cytoplasm. This population exhibited a tendency to colonize and replace preexisting germinal centers and was associated with a dense inflammatory infiltrate of T lymphocytes. The immunophenotypic profile of the tumor cells was as follows: CD20+, CD79a+, CD10‐, BCL6‐, BCL2+, CD23‐, CD5‐, Cyclin D1‐, with a Ki‐67 proliferation index of 10% (Figure 3). The patient underwent an inpatient osteomedullary biopsy, with no evidence of lymphoproliferative disease, and an esophagogastroscopy, which yielded negative results. The PET scan confirmed the presence of pelvic and subdiaphragmatic lymph nodes with high metabolic activity (Figure 4). Based on the collective findings, the patient was diagnosed with extranodal marginal zone lymphoma (EMZL) of mucosa‐associated lymphoid tissue (MALT), classified as Stage IV (Ann Arbor staging system) [9].

**FIGURE 3:**
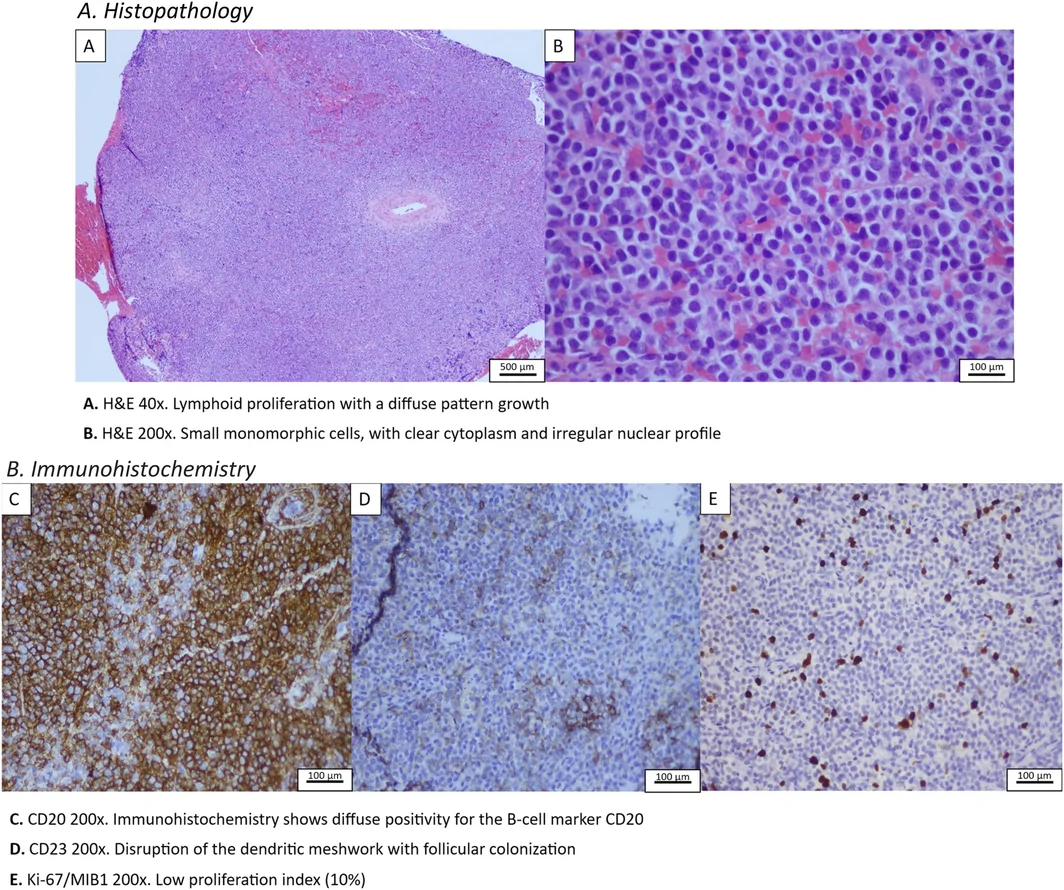
Extranodal marginal zone lymphoma (EMZL) of mucosa‐associated lymphoid tissue (MALT) on cervical and vaginal biopsies. (A) Histopathological features. (B) Immunohistochemical features.

**FIGURE 4:**
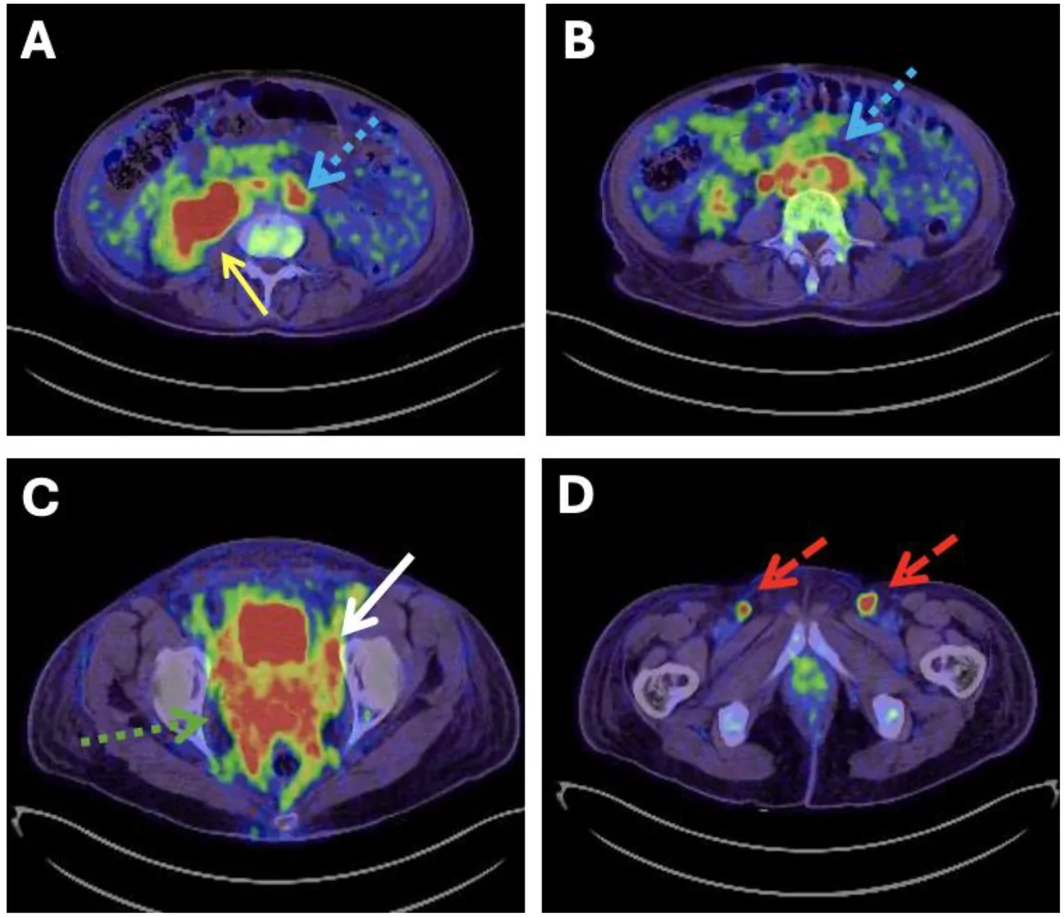
PET scan. (A, B) Demonstrate metabolically active lymphadenopathies in the lumbar‐aortic regions (light blue dotted arrows). Additionally, (A) reveals radiotracer accumulation within the dilated right urinary collecting system (yellow arrow), indicating obstruction of the excretory tract. (C) Highlights a large pelvic mass with intense FDG uptake, encasing the pelvic viscera (green dotted arrow), as well as metabolically active lymph nodes in the left iliac‐obturator chain (white arrow). (D) Shows bilateral inguinal lymphadenopathies with increased metabolic activity (red dashed arrows).

Considering the rarity and stage of the disease, the need to initiate immediate systemic treatment for symptom relief and disease control, as well as the indolent histotype unresponsive to conventional chemotherapy treatments, the patient was offered enrolment in an experimental study, which was accepted. The therapeutic regimen consisted of molecularly targeted therapies and immunotherapy, specifically a tyrosine kinase inhibitor and an anti‐CD20 monoclonal antibody. The study involved an induction phase with the anti‐CD20 monoclonal antibody in combination with a tyrosine kinase inhibitor, followed by a maintenance phase with the tyrosine kinase inhibitor alone.

The patient started induction therapy in February 2023.

Tumor marker levels were assessed at 1 month (March 2023) and 4 months (June 2023) after the initiation of treatment (Table 1).

CA 125 was the only tumor marker elevated at the time of diagnosis and showed a gradual decline throughout the course of therapy. In contrast, LDH levels were within the normal range at diagnosis but progressively increased, likely because of treatment‐related cytolysis.

After 2 months of treatment, a chest‐abdomen CT scan performed in April 2023 (Figure 5), revealed the resolution of pleural effusion and right hydronephrosis and the reduction in the pelvic mass (112 × 155 × 90 mm vs. 190 × 160 × 140 mm) and nodal lesions (para‐aortic: 13 × 10 mm vs. 16 × 12 mm; right external iliac: 12 × 8 mm vs. 23 × 16 mm; left inguinal: 13 × 7 mm vs. 23 × 16 mm; right inguinal: 15 × 8 mm vs. 25 × 15 mm).

**FIGURE 5:**
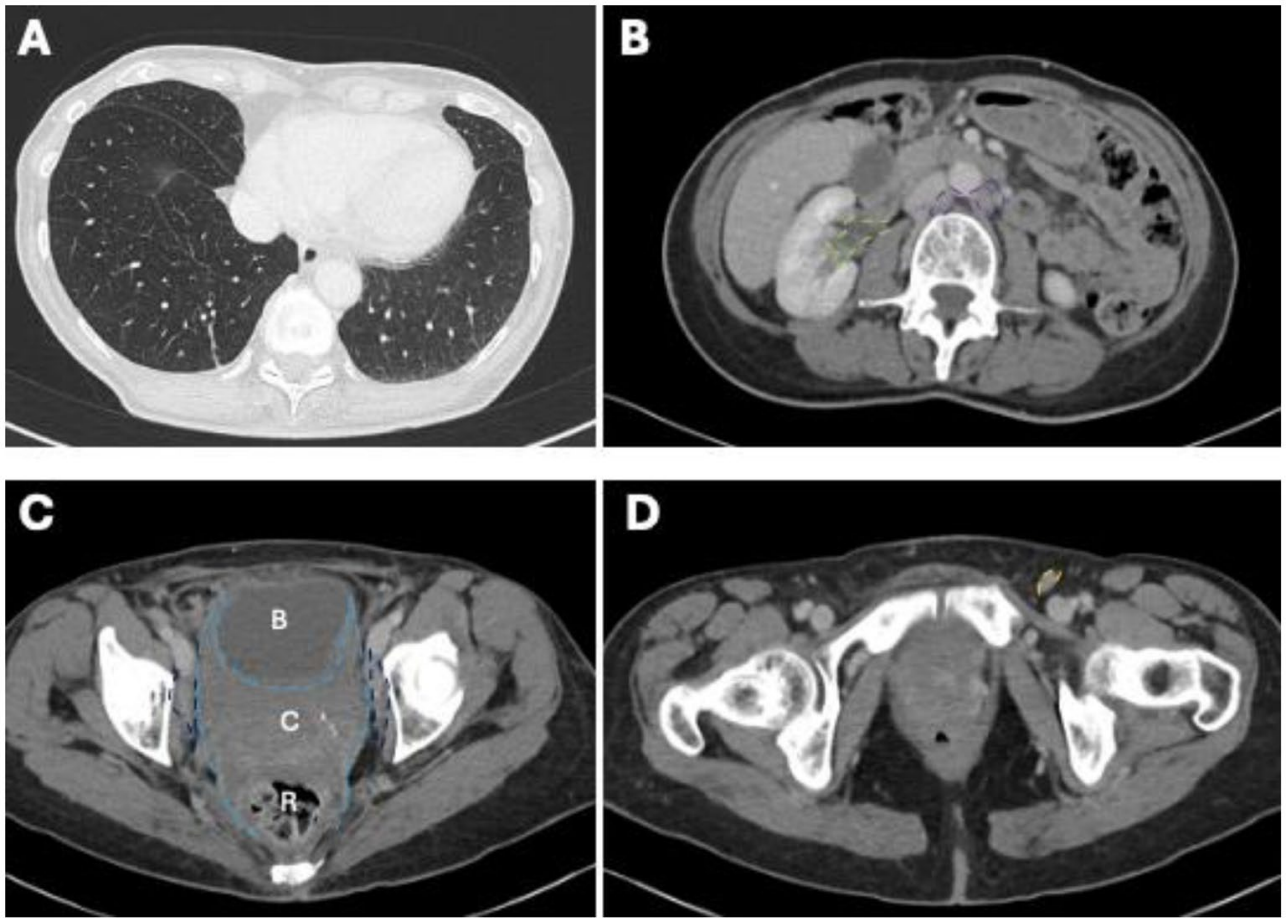
Chest‐abdominal CT scan after 2 months of treatment (April 2023). (A) Demonstrates complete resolution of the pleural effusion. (B) Reveals resolution of the right hydronephrosis (green dashed lines) as well as a reduction in para‐aortic lymphadenopathies (purple dashed lines). (C) There is a noticeable decrease in the size of the pelvic mass (light blue dashed lines) and iliac‐obturator lymphadenopathies (dark blue dashed lines). (D) Shows a reduction of inguinal nodes (orange dashed lines). Additionally, no ascitic fluid is observed.

A follow‐up CT scan performed after 6 months of treatment (August 2023) demonstrated a further reduction in the size of the pelvic mass (maximum diameters 64 × 90 × 70 mm) and shrinkage of all lymph nodes, all measuring less than 12 mm (Figures 6 and 7).

**FIGURE 6:**
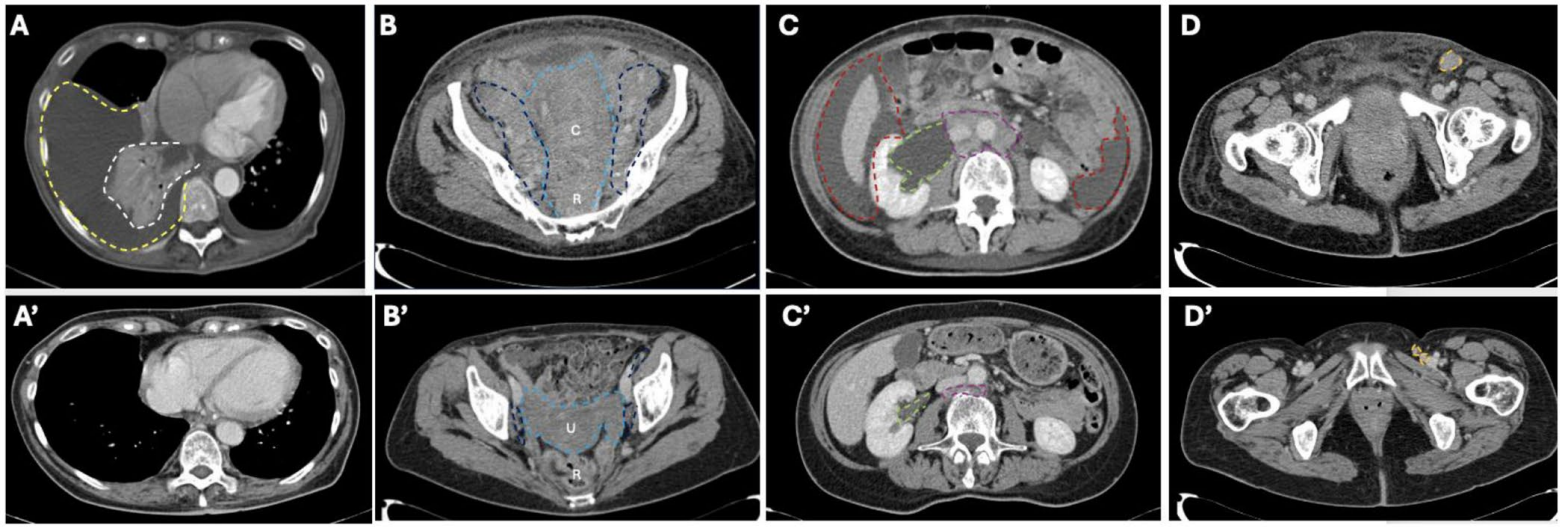
Chest‐abdominal CT scan after 6 months of treatment (August 2023). (A–D) Represent the CT scans before therapy, while A′–D′ correspond to the CT scans after 6 months of experimental therapy (August 2023). (A′) Illustrates complete resolution of the right pleural effusion (yellow dashed lines in A) and subtotal atelectasis (white dashed lines in A). (B′) Compared to B, the pelvic mass shows a significant decrease in size (light blue dotted arrows), with improved delineation of the pelvic organs, particularly the rectum, which now presents clear cleavage planes from the mass. It is also notable a significant reduction in iliac‐obturator lymphadenopathies (dark blue dashed lines). (C′)Relative to C, demonstrates a marked reduction in lumbar‐aortic lymphadenopathies (purple dashed lines), disappearance of right hydronephrosis (green dashed lines), and resolution of ascites (red dashed lines). (D′) Highlights a significant reduction in bilateral inguinal lymphadenopathies (orange dashed lines), which are clearly evident in D.

**FIGURE 7:**
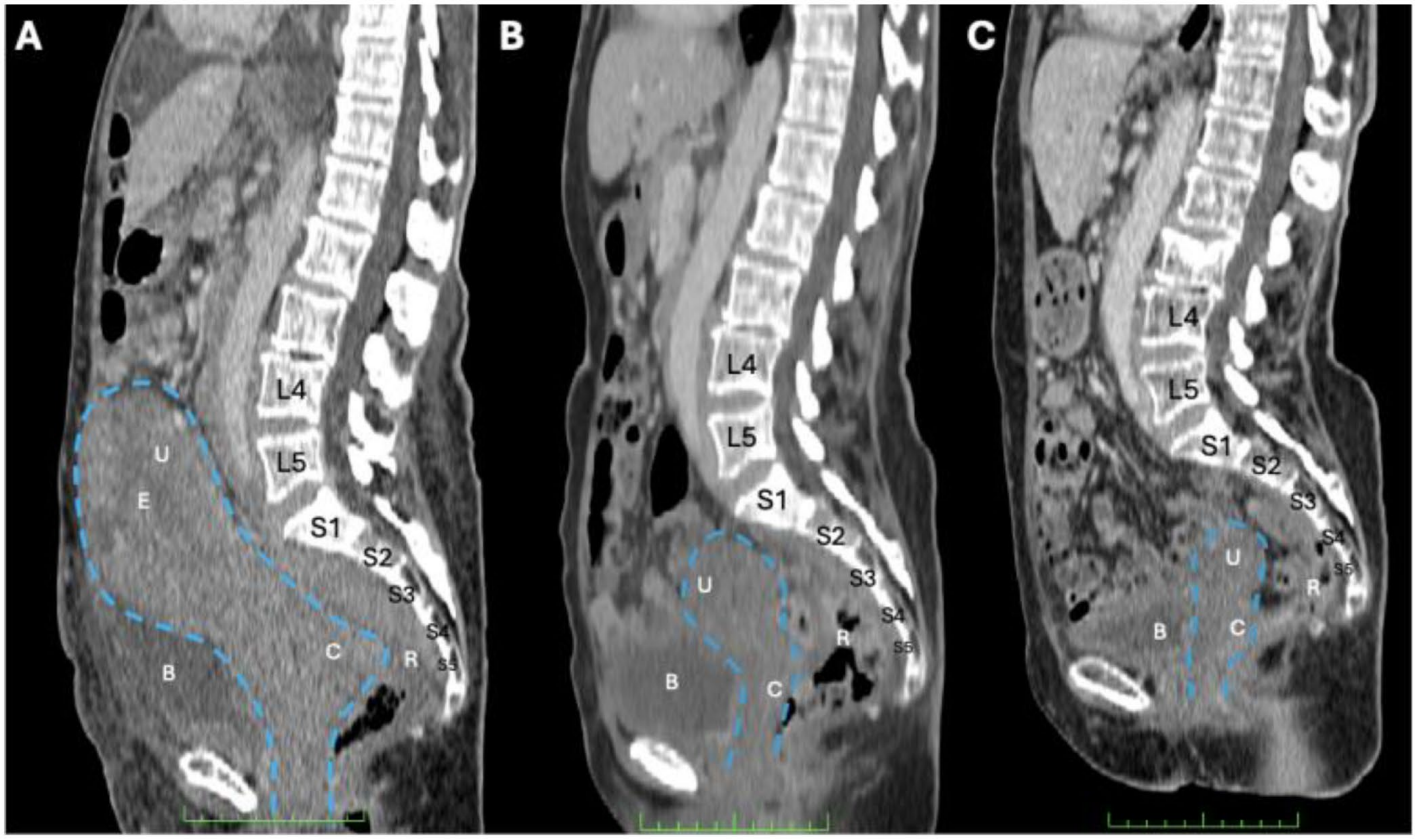
CT Imaging comparison before, after two and 6 months of experimental therapy, sagittal views. Multiplanar sagittal reconstructions at the time of diagnosis (A), after 2 months of therapy (B), and after 6 months of therapy (C). (A) Shows the enlarged uterus (U) outlined by a light blue dotted line, with the uterine fundus extending up to the umbilical line at the level of the superior endplate of L4. Note the distension of the endometrial cavity (E) and the absence of clear cleavage planes with the rectum (R) and bladder (B). (B) Reveals a marked reduction in uterine size, indicating significant regression in uterine volume following treatment; however, limited cleavage planes with the bladder (B) and rectum (R) are still evident. The superior margin of the uterus reaches the superior endplate of S1. The distension of the endometrial cavity is no longer present. (C) Demonstrates a further and significant response to therapy, with continued reduction of uterine volume; the uterine fundus reaches the level of the endplate of S3. Note the clearly visible cleavage plane between the uterus (U) and the rectum (R).

After 30 months from the start of therapy (August 2025), the patient is under maintenance treatment with maintained partial response and good performance status and resolution of initial symptoms and is currently in the maintenance phase of the experimental trial.

The clinical course of the patient, including diagnosis, treatment, radiological response, and follow‐up through August 2025, is summarized in Table 2.

**TABLE 2:** Clinical timeline of the patient.

Event	Date	Description
Diagnosis	January 2023	Stage IV MZBCL of the uterus diagnosed after cervical and vaginal biopsies
Treatment	February 2023	Phase 1: 8 induction cycles (completed in August) Phase 2: Maintenance therapy (up to 2 years)—Ongoing
Radiological response	April–August 2023	Reduction of the pelvic mass and enlarged lymph nodes in the abdominopelvic region
Last follow‐up	August 2025	Maintenance of partial response

## 3 Literature Review

A literature search was conducted using the PubMed, Scopus, and Web of Science databases to identify case reports and case series of primary marginal zone B‐cell lymphoma (MZBCL) of the cervix and uterine corpus up to 2023 to analyze their characteristics. The search was performed using combinations of the following keywords: “MALT lymphoma,” “extranodal marginal zone lymphoma,” “uterine cervix,” “cervix,” “uterine corpus,” and “endometrium.”

For the generation of Table 3, we excluded cases with inadequate clinical and therapeutic information and those in which the primary nature of the lymphoma was uncertain.

**TABLE 3:** Clinical studies reporting cases of EMZL involving the uterine cervix and corpus.

Authors	Article year	Age range	Clinical presentation	Sites	Histologic type	Stage	Treatment
Pelstring et al. [10] (*n* = 1)	1991	40	Vaginal bleeding, uterine leiomyoma	Cervix	MZBCL	IIE	S‐RT
Ballesteros et al. [11] (*n* = 1)	1998	72	Abdominal pain	Corpus	MZBCL	IIE	S‐RT
Vang et al. [12] (*n* = 10)	2000	35–67	Cervical dysplasia, vaginal bleeding	Corpus (1) cervix (9)	DLBCL (8) FL (1) MZBCL (1)	IE (7) IIE (3)	S (3), CT‐RT (3) S‐CT‐RT (2), CT (1) S‐CT (1)
Rossi et al. [13] (*n* = 1)	2001	46	Intermenstrual vaginal bleeding, endocervical polyp	Cervix	MZBCL	IVE	CT‐S
Iyengar et al. [14] (*n* = 1)	2004	65	Postmenopausal bleeding	Corpus	MZBCL	IE	S
Kosari et al. [6] (*n* = 40)	2005	N.R.	N.R.	Corpus (16) Cervix (24)	DLBCL (24) BL (4), FL (7), MZBCL (2), HL‐LD (1), PBLL (2)	N.R. (40)	N.R. (40)
Frey et al. [15] (*n* = 4)	2006	35–56	Menorrhagia post‐menopausal vaginal bleeding	Cervix (3) Corpus (1)	DLBCL (3) MZBCL (1)	IIE (4)	CT‐S‐ CT (1), S‐CT (2), S (1)
Hamadani et al. [16] (*n* = 1)	2006	52	Vaginal bleeding	Corpus	MZBCL	IVE	S
Coon et al. [17] (*n* = 1)	2008	56	Vaginal spotting	Cervix	MZBCL	N.R.	S‐RT
Heeren et al. [18] (*n* = 1)	2008	61	Incidental finding after vaginal prolapse	Corpus	MZBCL	IE	S
Annibali et al. [19] (*n* = 1)	2009	81	Incidental finding in an endometrial polyp	Corpus	MZBCL	IE	None
De Angelis et al. [20] (*n* = 1)	2011	55	Polyp of the corpus uteri	Corpus	MZBCL	IE	S
Di Tucci et al. [21] (*n* = 1)	2013	55	Abnormal uterine bleeding, endometrial polyp	Corpus	MZBCL	IE	S
Sugimoto et al. [22] (*n* = 1)	2013	72	Abdominal fullness	Cervix	DLBCL (transformed from MZBCL)	N.R.	CT‐S
Merrit et al. [23] (*n* = 1)	2014	77	Utero‐ vaginal prolapse, urinary frequency and nocturia	Corpus	MZBCL	IE	S
Tahmasebi et al. [24] (*n* = 4)	2015	46–72	Vaginal bleeding	Corpus (4)	MZBCL (4)	IE (4)	None (4)
Tomoko Takimoto et al. [25] (*n* = 1)	2017	71	Vaginal bleeding	Cervix	MZBCL	IIE	CT
Zhang et al. [26] (*n* = 1)	2019	61	Postmenopausal vaginal bleeding for 2 months	Corpus	MZBCL	IE	S
Weng et al. [27] (*n* = 13)	2023	22–74	Abnormal vaginal bleeding, abdominal pain	Cervix (13)	DLBCL (12) MZBCL (1)	IE (8) IIE (2) IVE (3)	S‐ RT‐ CT (1) CT (6), RT (1), CT‐RT (2), C‐S (2), S‐C (1)
This case	2024	65	Dyspnoea, massive oedema of the lower extremities	Corpus/cervix	MZBCL	IV	Experimental trial
Forooshani et al. [28] (*n* = 9)	2026	20–83	Abnormal vaginal bleeding, abdominal pain	Cervix (8) Corpus (1)	DLBLC (5) FL (2) LL (1) MZBCL (1)	N.R	N.R.

Abbreviations: BL, Burkitt's lymphoma; CT, chemotherapy; DLBCL, diffuse large B‐ cell lymphoma; FL, follicular lymphoma; HL, Hodgkin lymphoma; MZBCL, marginal zone B‐cell lymphoma; NR, not reported; PBLL, precursor B‐lymphoblastic lymphoma; RT, radiotherapy; S, surgery.

Diffuse large B‐cell lymphoma is the most common histological subtype of gynPELs [12]. The most frequently involved organ remains a topic of debate, although the ovary is most commonly reported [29]. The origin of uterine lymphoma remains unknown, although a potential association with chronic inflammation has been suggested [12, 29]. Patients typically have access to primary care with non‐specific symptoms, such as abnormal gynaecologic bleeding, which can be misdiagnosed as other common uterine conditions. Although there is no consensus on the management of primary uterine lymphoma, previous studies have discussed various treatment approaches, primarily through narrative descriptions. Treatment options should align with the guidelines for primary extranodal lymphoma (PEL). Chemotherapy and/or radiotherapy remain the cornerstone of treatment for extranodal lymphomas, with specific regimens and dosages depending on the histological subtype [30]. Primary uterine localization of EMZL has been described only anecdotally: we identified approximately 24 cases in the literature. The median age of patients was 57 years (range 40–81). The clinical presentation reported in the review included abnormal vaginal bleeding in most cases (*n* = 15), followed by incidental findings (*n* = 5), abdominal fullness (*n* = 1), and abdominal pain (*n* = 1). In most cases, the lymphoma was confined to the uterus. However, three cases showed local nodal involvement, and one case presented with bone marrow localization. Other significant findings included uterine leiomyomas (*n* = 2), endometrial polyps (*n* = 3), endocervical polyp (*n* = 1), hypothyroidism (*n* = 1), and cancer (*n* = 1). In the literature, we identified one report describing a primary MALT lymphoma involving the ovary, fallopian tube, and appendix in the context of endometriosis, suggesting that inflammation may still play a role in select cases [31].

## 4 Discussion

Primary extranodal marginal zone B‐cell lymphoma (MZBCL) of the uterus remains an exceptionally rare entity, and its diagnosis is frequently delayed because of the overlap between clinical presentation and more common gynaecologic malignancies. The present case highlights several clinically relevant findings.

First, as reported in the literature review, uterine EMZL may present with non‐specific systemic manifestations rather than typical gynaecological symptoms. Unlike most previously reported uterine MZBCL cases, which commonly presented with abnormal vaginal bleeding and were diagnosed at localized stages, our patient presented with dyspnea, massive lower limb oedema, pleural effusion, hydronephrosis, bulky pelvic disease, and disseminated nodal involvement. This atypical presentation initially suggested advanced ovarian cancer rather than lymphoma. Moreover, despite extensive disease burden, bone marrow involvement was absent, emphasizing the heterogeneous dissemination pattern of EMZL.

Second aspect of relevance is that imaging findings that may strongly mimic advanced gynaecologic malignancies, leading to an initial diagnostic suspicion of ovarian or uterine sarcoma‐like disease. Our findings are partially consistent with previously published reports regarding the diagnostic difficulties associated with female genital tract lymphomas. As described in the literature review presented in Table 3, lymphomas of the cervix typically arise in the stroma, making cervical smears often negative. Diagnostic capability at later stages is obtained after significant tissue destruction, with low specificity due to the high incidence of benign lymphoid aggregates in these samples [32]. The typical presentation of primary malignant lymphoma of the cervix is a diffuse, submucosal, bar‐shaped enlargement of the cervix, with routine cervical smears frequently yielding negative results [33]. Extranodal marginal zone B‐cell lymphoma (EMZL) is a subtype of indolent non‐Hodgkin lymphoma that develops in extranodal organs and constitutes approximately 7%–8% of NHL cases [34]. Although traditionally considered a localized disease, dissemination can occur, spreading within the MALT system, bone marrow, and lymph nodes after the loss of the properties related to mucosal homing [35]. The differential diagnosis of MALT lymphomas of the endometrium includes inflammatory processes such as endometritis, which may be associated with infections like Chlamydia. Differently, in chronic endometritis, the cells' population is represented by plasma cells, lymphocytes, macrophages, and neutrophils.

The imaging characteristics observed in this case also deserve attention. MRI and CT demonstrated massive uterine enlargement, distension of the endometrial cavity, diffuse infiltration of surrounding pelvic structures, and extensive lymphadenopathy. These findings may overlap with advanced gynaecologic malignancies; however, some features—including diffuse submucosal enlargement of the cervix, preservation of tissue planes despite extensive compression, and discordance between tumor bulk and limited mucosal abnormalities—may raise suspicion for lymphoma. Recognition of these patterns may facilitate earlier biopsy‐driven diagnosis.

Third aspect is that timely tissue sampling and multidisciplinary evaluation are essential to establish the diagnosis and avoid unnecessary radical surgery. Interdisciplinary collaboration among gynecologists, pathologists, and hematologists is crucial for the optimal management of affected patients.

The strengths of this report include the detailed longitudinal radiological documentation, long‐term follow‐up, comprehensive multidisciplinary diagnostic pathway, and description of response to an innovative therapeutic strategy. Additionally, this case contributes to the limited evidence regarding disseminated uterine EMZL and expands the spectrum of possible clinical presentations. Limitations are related to the single‐case nature of the report; nonetheless, the experimental treatment that is both elements that may limit the comparisons of the case to more common conditions.

Overall, this case reinforces the importance of maintaining lymphoma in the differential diagnosis of large pelvic masses with atypical clinical features. Early biopsy, multidisciplinary discussion, and avoidance of upfront radical surgery may improve diagnostic accuracy and facilitate timely initiation of appropriate systemic treatment.

## 5 Conclusions

Primary uterine extranodal marginal zone lymphoma is a rare and diagnostically challenging malignancy that may mimic advanced gynaecologic cancers. The present case is notable for its unusual presentation with systemic symptoms, extensive pelvic involvement, and disseminated disease despite an indolent histology. Histopathological confirmation of EZML is essential to establish the diagnosis and avoid unnecessary surgery. The patient had no history of endometritis or cervicitis, a finding consistent with other reported cases. While MALT lymphomas in other anatomical sites (the thyroid in Hashimoto's thyroiditis and the stomach in chronic atrophic gastritis associated with *Helicobacter pylori* ) are clearly linked to chronic inflammatory processes, this association has not been observed in the female genital tract.

This case highlights the importance of maintaining a broad differential diagnosis, the value of multidisciplinary collaboration, and the potential role of experimental therapies in the management of rare and complex diseases.

## Author Contributions

**Valeria Matera:** conceptualization, investigation, writing – original draft, funding acquisition, data curation. **Laura Bandiera:** methodology, funding acquisition. **Manuela Bramerio:** methodology, funding acquisition. **Maria Deodato:** data curation. **Carmela Quatrale:** data curation. **Liliana Marchetta:** data curation. **Mariachiara Palucci:** data curation. **Tommaso Bignardi:** data curation. **Maria Lieta Interdonato:** data curation. **Giuseppe Franco Cicala:** investigation, data curation. **Federico Spelzini:** data curation. **Lorenzo Ceppi:** project administration, validation, supervision, conceptualization.

## Funding

The authors have nothing to report.

## Ethics Statement

The presented report was approved by the Local Ethic Committee (Registry opinion no. 135, approved on 17 February 2023).

## Consent

Written informed consent was obtained from the patient for publication of this case report and accompanying images.

## Conflicts of Interest

The authors declare no conflicts of interest.

## Data Availability

The data that support the findings of this study are available from the corresponding author upon reasonable request.
